# Factors Affecting Pain Control in Patients with Sickle Cell Disease at Mwananyamala and Muhimbili Hospitals in Dar es Salaam, Tanzania

**DOI:** 10.3390/jcm15062339

**Published:** 2026-03-19

**Authors:** Happiness Joseph Igogo, Mbonea Yonazi, Ritah F. Mutagonda, Avelina Mgasa, Mwashungi Ally, Clara Chamba, Ahlam Nasser, William Mawalla, Magdalena A. Lyimo, Benson Kidenya, Agness Jonathan, Florence Urio, Paschal Rugajjo, Emmanuel Balandya, Lulu Chirande

**Affiliations:** 1 Department of Haematology and Blood Transfusion, Muhimbili University of Health and Allied Sciences, Dar es Salaam P.O. Box 65001, Tanzania; mwashungi.ally@muhas.ac.tz (M.A.); cchamba@blood.ac.tz (C.C.); dr.ahlamnasser@gmail.com (A.N.); mawallawf65@gmail.com (W.M.); magdalena.lyimo@gmail.com (M.A.L.); 2 Sickle Pan-African Research Consortium (SPARCO), Dar es Salaam P.O. Box 65001, Tanzania; myonazi@gmail.com (M.Y.); rittdavisrida@yahoo.com (R.F.M.); benkidenya@yahoo.com (B.K.); ajonathan@blood.ac.tz (A.J.); furio@gmail.com (F.U.); prugajjo@gmail.com (P.R.); ebalandya@yahoo.com (E.B.); chirandelulu@yahoo.com (L.C.); 3 Muhimbili National Hospital, Dar es Salaam P.O. Box 65000, Tanzania; 4 Department of Clinical Pharmacy and Pharmacology, Muhimbili University of Health and Allied Sciences, Dar es Salaam P.O. Box 65013, Tanzania; 5 National Blood Transfusion Services, Dar es Salaam P.O. Box 65019, Tanzania; amgasa@gmail.com; 6 Department of Biochemistry and Molecular Biology, Catholic University of Health and Allied Sciences, Mwanza P.O. Box 1464, Tanzania; 7 Department of Biochemistry and Molecular Biology, Muhimbili University of Health and Allied Sciences, Dar es Salaam P.O. Box 65001, Tanzania; 8 Department of Physiology, Muhimbili University of Health and Allied Sciences, Dar es Salaam P.O. Box 65001, Tanzania; 9 Department of Pediatric and Child Health, Muhimbili University of Health and Allied Sciences, Dar es Salaam P.O. Box 65001, Tanzania

**Keywords:** sickle cell disease, pain severity, pain control, hospital, Tanzania

## Abstract

**Background/Objective**: The most common hemoglobin disorder in the world is SCD. The majority of SCD cases come from Africa, accounting for up to two-thirds of the 300,000 annual births of individuals with SCD worldwide. In Tanzania, 11,000–14,000 babies are born with SCD each year. Despite treatment advancement, pain is still an attributable cause of admissions among patients with SCD. However, data are still lacking regarding the adequacy of pain control in patients with SCD in Tanzania. The aim of this study was to determine factors affecting pain control among patients with SCD presenting with painful events at Mwananyamala Regional Referral Hospital (MRRH) and Muhimbili National Hospital (MNH) in Dar es Salaam, Tanzania. **Methods**: This was a cross-sectional study conducted at MRRH and MNH, which are tertiary referral hospitals in Dar es Salaam, Tanzania. Patients with SCD aged 8 years and above who presented at the hospitals with painful events (from August 2022 to February 2023) were enrolled in the study. We used a structured questionnaire to collect data on participants’ socio-demographic characteristics and clinical parameters. The adequacy of pain control was assessed using the WHO Pain Management Index. Multivariable binary logistic regression was used to determine factors associated with pain control. Differences were considered statistically significant when the *p*-value was <0.05. **Results**: A total of 390 patients with SCD were analyzed. The mean age (±SD) was 15 (±6) years. Most patients were recruited from outpatient clinics (88.2%). The male-to-female ratio was 1:1. The majority of patients had less than three pain episodes per year (77.9%), and about 64.6% presented to the hospital with mild pain. The proportion of patients on hydroxyurea was 62.3%. Furthermore, one-third of patients had inadequate pain control. Factors associated with inadequate pain control included receiving initial pain management in other health facilities (adjusted odds ratio [aOR] and 95% confidence interval [CI] = 2.5 (1.5–4.5), *p* = 0.001), presenting to the hospital with moderate pain (aOR = 2.2, 95% CI [1.3–3.8], *p* = 0.0060), and presenting to the hospital with a fever (aOR = 3.8, 95% CI [1.1–13.9], *p* = 0.04). Having severe pain and receiving initial treatment at MRRH and MNH seemed to be protective factors (aOR = 0.33, 95% CI [0.11–0.97], *p* = 0.04, and aOR = 0.29, 95% CI [0.14–0.61], *p* = 0.001, respectively). **Conclusions**: A considerable proportion of patients with SCD receive suboptimal pain control. Receiving initial pain management from another healthcare facility, presenting to the hospital with moderate pain, and having a fever were associated with inadequate pain control. Further research is warranted to elucidate ways of optimizing the management of pain in patients with SCD in Tanzania.

## 1. Introduction

Sickle cell disease (SCD) is a genetic disorder of the red blood cells (RBCs) resulting from a point mutation substituting valine for glutamic acid at position six of the β-globin chain [1,2]. Sickle cell-related painful crises and hemolytic anemia are the most common reasons for hospital admission. Repeated VOC in SCD results in complications in different organ systems that lead to lifelong disabilities [3,4,5]. In Tanzania, 11,000–14,000 children are born with SCD each year [6,7].

Pain in SCD remains the leading cause of hospitalization [8]. Pain in patients with SCD typically occurs from the first year of life, concomitant with the fall in levels of fetal hemoglobin. Unlike sickle hemoglobin (HbS), fetal hemoglobin (HbF) can resist sickling when subjected to changes such as hypoxia, which usually triggers pain; hence, its presence in high levels protects against sickling [6]. Pain in patients with SCD varies from acute to chronic to neuropathic pain [6,9]. Pain control refers to the act of managing discomfort to a tolerable level. This can be achieved through the use of anti-pain medications and other non-pharmacological means, such as pain distraction, e.g., watching TV.

Despite advances in treatment and the introduction of disease-modifying drugs such as hydroxyurea, pain remains the most common cause for hospitalization among SCD patients [1,7,10]. Studies have indicated that patients with SCD frequently suffer from both undertreatment and overtreatment of their pain in hospitals [11,12,13]. Adequate pain control involves several measures, including adequate hydration and selecting suitable pain medication based on the World Health Organization (WHO) analgesic ladder, as well as treating any underlying causes of pain if present [14]. Analgesia should be administered to SCD patients within 30 min of hospital admission, and patients should be reviewed after receiving analgesia to assess its effectiveness [4,6,9,15]. Adequate pain control is attainable if suitable analgesia is used at the proper dose and frequency and is administered according to the WHO analgesic ladder alongside proper management of hydration [9]. According to the WHO, there are three levels of pain: mild pain, moderate pain, and severe pain [2,6]. The appropriate analgesia for mild pain (level one) is a non-opioid analgesia, such as paracetamol. When it is not enough, a non-steroid anti-inflammatory drug (NSAID), adjuvant therapy such as massage, and pain distraction therapy such as television are used. Level two pain (mild to moderate) is managed by weak opioids such as codeine plus or minus non-opioid medication, and level three (severe pain) is managed by strong opioids such as morphine [4,7].

The adequacy of pain management among patients with SCD in Tanzania is yet to be fully explored. This study aimed to explore patients’ experiences and factors associated with adequate pain control among patients with SCD in Dar es Salaam, Tanzania, to inform measures for the optimization of pain management in this patient population.

## 2. Methodology

### 2.1. Study Design and Study Setting

This was a cross-sectional study conducted at Mwananyamala Regional Referral Hospital (MRRH) and Muhimbili National Hospital (MNH) in Dar es Salaam, Tanzania. MNH is the only national hospital in Tanzania, located in the Ilala district, and offers SCD-integrated care to adult and pediatric patients, attending to about 100–150 patients per week. The clinics are held on Thursdays for pediatrics and Fridays for adults at the general hematology clinics. The hospital also offers inpatient care, and about 10–20 patients with SCD are admitted monthly; nevertheless, emergency services are available for those presenting with acute SCD pain. The hospital has adequate physicians, hematologists, pediatricians, nurses, and pharmacists who provide specialized care to all patients with SCD. MRRH is a tertiary-level hospital located in Kinondoni district and provides integrated SCD care, with regular SCD outpatient clinics on Tuesdays, attending to about 30–40 patients with SCD per week. The hospital is capable of providing emergency services and inpatient care.

Both of these hospitals receive referral cases from other lower-level hospitals; however, some patients come directly from home for their regular clinic visits. Hydroxyurea is readily available for patients attending these two hospitals, and all important paid medications, including opioid analgesics, NSAIDs, and paracetamol, are available.

MRRH and MNH are part of the Sickle Pan Africa Research Consortium (SPARCO) Tanzania project, which maintains an electronic registry for patients with SCD who are regularly seen at these hospitals and 12 other health facilities across four administrative regions in Tanzania.

### 2.2. Sample Size and Sampling Method

Study participants were all patients with SCD, aged 8 years and above, who presented at MRRH and MNH with painful events from August 2022 to February 2023.

The sample size for this study was obtained using the formula described by Leslie Kish.

*n* = z^2^p(1 − p)

e^2^

Z = level of confidence (1.96 for 95% confidence level), ε = margin of error, which is 5%, and p = proportion with adequate pain control, which was taken as 50% due to the lack of similar studies that assess the adequacy of pain control in patients with SCD.

*n* = 1.96^2^ × 50(100 − 50)

5^2^

*n* = 384

Participants in the study were recruited consecutively via purposive sampling from clinics and inpatient wards until the desired sample size of 384 individuals was attained.

### 2.3. Data Collection Methods

Data was collected at MRRH and MNH by the principal investigator (PI) and a trained research assistant. For patients attending SCD clinics, the PI explained the study and inquired whether they were experiencing sickle cell-associated pain that day or not. Only those who had pain and consented were enrolled in the study. For inpatients, the attending nurses and clinicians were informed about the study and notified the PI via phone whenever patients with SCD pain were admitted. The PI then attended to the patients and informed them about the study and its advantages, obtained written consent/assent, and enrolled patients who agreed to participate in the study. Patients who were in a critical condition, such as those admitted to the intensive care unit (ICU), those with documented neurological conditions such as dementia, cognitive impairment, or psychosis, and those with other identifiable causes of pain apart from SCD, were excluded from this study. A physical examination and hydration status assessment were also performed on each patient, and vital signs were taken, including pulse rate, blood pressure, and temperature. Other clinical characteristics were obtained from the patients’ medical records (SCD Health Passport).

A structured questionnaire was used to collect data. The questionnaire had three parts: The first part collected data on socio-demographic characteristics of the patient, including the patient’s number (code), date of birth, gender, weight, residence, marital status, education level, number and frequency of admissions due to painful events, steady-state hemoglobin, weight, and medication history. The second part incorporated the standardized age-appropriate pain assessment tool, where level 0 was interpreted as no pain, levels 1–3 were mild pain, levels 4–7 were moderate pain, and levels 8–10 were interpreted as severe pain. For pediatrics and young adults, we used the Numeric Pain Rating Scale, while in adults aged 18 years and above, the Brief Pain Inventory Scoring Short Form was used [16,17]. The third part of the questionnaire recorded the hydration status and adequacy of pain control, which was assessed within 12–72 h from the first dose of analgesia using the Pain Management Index. Any negative number on the Pain Management Index indicated inadequate pain control; 0 and numbers above it (positive numbers) were interpreted as appropriate management for the level of pain [16]. The Pain Management Index measures the adequacy of pain control by taking into account the treatment and the intensity of pain.

### 2.4. Statistical Analysis

The collected data were sorted and approved for completeness and consistency. Data were de-identified, coded, cleaned, and entered into Statistical Package for Social Sciences (SPSS) version 23 for analysis. The clinical characteristics and laboratory parameters of patients presenting with pain were analyzed using a frequency distribution table and percentages. The proportion of patients with SCD who had adequate pain control was obtained. The association between the adequacy of pain management and the independent variables was determined by cross-tabulation using Pearson’s chi-square or Fisher’s exact test. All factors with a *p*-value < 0.2 in the bivariate analysis were entered into a multivariable binary logistic regression analysis to determine the independent factors associated with inadequate pain control. Results were considered statistically significant when the *p*-value was less than 0.05.

### 2.5. Ethical Consideration

This study received ethical approval from the Muhimbili University of Health and Allied Sciences (MUHAS) Institutional Review Board (IRB), number “MUHAS-REC-10-2022-1420”, as well as separate permissions from MRRH and MNH. Written informed consent/assent was obtained from each participant. No participant names or identities were recorded; each study participant was coded using numbers. De-identification of data during the process of data entry was performed to minimize the association of patients with their data. Only individuals directly involved with the research had access to the data. Patient confidentiality was further enhanced by storing all the collected information in a locked cabinet and in a computer with a secure password.

## 3. Results

### 3.1. Socio-Demographic and Clinical Characteristics of the Study Participants

**Figure 1:**
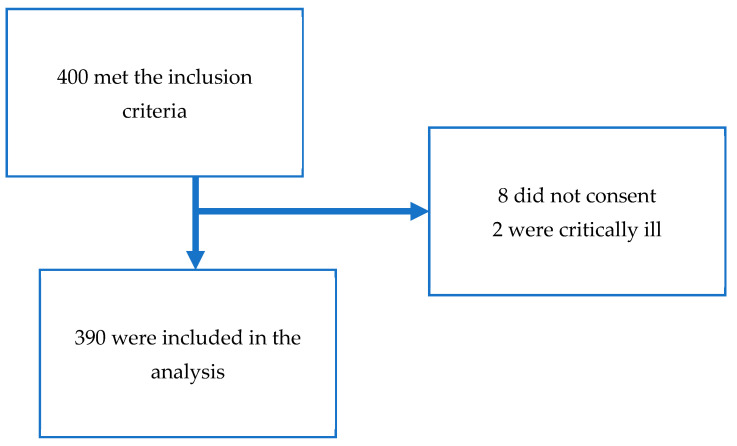
Flowchart diagram of patients enrolled in the study.

A total of 400 patients with SCD met the inclusion criteria. Of these, 10 were excluded as follows: two were admitted to the ICU, where one experienced convulsions that led to sedation; another had pain due to a skin reaction secondary to antibiotic use (amoxicillin); and eight patients did not consent to participate in the study (Figure 1).

Most of the study participants, 344/390 (88.2%), were enrolled from outpatient settings. The mean age of the participants was 15 years, and there was a balance between males and females (1:1 ratio). Further, 166/390 (42.6%) participants were residing outside of Dar es Salaam. Over two-thirds of participants, 304/390 (77.9%), experienced pain under three episodes annually, whereas 86/390 (22.1%) had three or more episodes. The majority of participants, 252/390 (64.6%), reported mild pain, and the remaining one-third suffered moderate to severe pain. Two-thirds of patients were on hydroxyurea, and 16 patients presented with fever. Out of the 16 patients who presented with fever, 10 were under 5 years of age (Table 1).

### 3.2. Factors Associated with Inadequate Pain Control

Among the SCD patients enrolled in our study, we found that 31% (121/390) had inadequate pain control. Upon bivariate analysis, there was more inadequate pain control among those aged above 14 years compared to those 14 years and below (36.9% vs. 27.0%, *p* = 0.04). Patients who lived outside Dar es Salaam were more likely to have inadequate pain control compared to those who lived in Dar es Salaam (40.4% vs. 24.1%, *p* = 0.001). Having moderate pain was associated with inadequate pain control compared to those with mild or severe pain (52.4% vs. 24.6% vs. 14.3%, *p* < 0.001). Patients who were initially treated at home or in other healthcare facilities were more likely to have inadequate pain control compared to those initially treated at MRRH/MNH (31.8% vs. 49% vs. 9.9%, *p* < 0.001). Furthermore, although not statistically significant, patients who were not on hydroxyurea and those who presented to the hospital with fever were more likely to have inadequate pain control compared to their counterparts (36.7% vs. 27.6%, *p* = 0.07, and 50% vs. 30.2%, *p* = 0.09, respectively) (Table 2).

### 3.3. Independent Factors Associated with Inadequate Pain Control

To ascertain independent predictors of inadequate pain control, we performed multivariable binary logistic regression analysis. Patients who lived outside Dar es Salaam were 74% more likely to have inadequate pain control compared to those who lived in Dar es Salaam (aOR = 1.74, 95% CI [1.1–2.9], *p* = 0.03) Presenting to the hospital with moderate pain increased the odds of inadequate pain control 2.2-fold compared to those presenting with mild pain (aOR = 2.2, 95% CI [1.3–3.8], *p* = 0.006). On the contrary, those who presented to the hospital with severe pain had reduced the odds of having inadequate pain control by 67% compared to presenting with mild pain (aOR = 0.33, 95% CI [0.1–1.0], *p* = 0.04). Patients presenting to the hospital with a fever were 3.8 times more likely to have inadequate pain control compared to those presenting with no fever (aOR = 3.8, 95% CI [1.1–13.9], *p* = 0.04). Compared to receiving initial treatment at home, receiving initial pain management at other healthcare facilities increased the odds of inadequate pain control 2.5-fold (aOR 2.5, 95% CI [1.5–4.5], *p* = 0.001). Receiving initial pain management at MRRH/MNH reduced the odds of having inadequate pain control by 71% (aOR 0.29, 95% CI [0.1–0.6], *p* < 0.001) (Table 3).

## 4. Discussion

To the best of our knowledge, this is the first study conducted using a large sample size to investigate factors affecting the adequacy of pain control among patients with SCD in Tanzania. We found that one-third of patients with SCD presenting at tertiary-level hospitals with pain had inadequate pain control. Residing outside Dar es Salaam and receiving initial treatment at hospitals other than the MRRH and MNH study sites, as well as presenting to the hospital with moderate pain or fever, were associated with inadequate pain control, while presenting with severe pain appeared to be a protective factor. This study provides critical insights into factors associated with the adequacy of pain control in one of the settings with the highest burden of SCD in sub-Saharan Africa and could inform measures to optimize pain management in this patient population.

Living outside Dar es Salaam was associated with an increased chance of inadequate pain control. The possible explanation for this observation is that there is more specialized care in Dar es Salaam than in other regions. However, further studies are needed to assess what factors contribute to this discrepancy. Studies have shown that adult patients with SCD are likely to visit multiple hospitals for treatment of their acute pain [18]. This could be the reason for seeking medical care at MRRH and MNH, despite receiving care in other hospitals.

We observed that patients who presented with moderate pain had twofold higher odds of having inadequate pain management. This could be because most patients were enrolled in the clinic and ended up receiving pain medication in an outpatient setting, where parenteral medication for pain and IV fluids are not normally administered. The initiation of outpatient infusion centers (ICs), where patients could receive IV fluids and parenteral pain medications, as is being conducted in other countries such as Uganda [6], will enable patients with moderate pain to receive parenteral medication, which will improve the adequacy of pain management in outpatient settings. The American Society of Hematology recommends that patients with SCD who present to the hospital with pain should be given analgesia within one hour of arrival at the hospital. It also recommends pain reassessment to be performed within 30 min after the initial dose of analgesia [19].

This study showed almost a fourfold increase in odds of inadequate pain control for patients who presented to the hospital with a fever. This association highlights infection as the major trigger for pain in patients with SCD and calls attention to proper management of infection for adequate pain relief to be achieved. A study conducted by Makani et al. at MNH found that fever was the second most common cause of admission, especially in children under 5 years of age who had SCD [7]. This was similar to the observation from our study, where nearly two-thirds of the patients who had fever were under 5 years of age. Receiving pain control at MRRH and MNH seemed protective in terms of adequacy of pain control as opposed to treatment received in other healthcare facilities. This could be due to the availability of specialized care and access to relatively more pain medications, including morphine, at MRRH and MNH.

The majority of patients with SCD who presented with painful events at MRRH and MNH attained adequate pain control. However, this study found that about one-third of patients had inadequate pain control. This was slightly lower compared to another study conducted in Uganda, which showed that a larger percentage of children (65.6%) who presented with SCD-related painful events had inadequate pain control [6]. The study in Uganda included only children attending outpatient SCD clinics, contrary to our study, which included both children and adults in both outpatient and inpatient settings. Further, two-thirds of patients reported having only mild pain. This could be because, by the time they arrived at MRRH and MNH, they had previously received pain medication either at home or at another healthcare facility, and only one-third of patients received their initial pain management at MRRH and MNH. A study conducted in Tanzania by Mkoka et al. indicated that patients with SCD, in coping with day-to-day painful events that are related to SCD, use “self-care remedies” for pain control [20]. This was similar to our study, where a significant number of patients self-medicated for pain at home. Patients who received pain medication at home were adequately managed compared to those who received pain medication at facilities other than MRRH and MNH. We speculate that this is because the pain treated at home is mostly mild pain, which can be controlled by over-the-counter pain medications such as paracetamol. Our study highlights the need to educate patients with SCD on home-based pain interventions for SCD pain [20].

Although individuals who were on hydroxyurea appeared more likely to have adequate pain control, this observation did not reach statistical significance in our study, despite the known effects of hydroxyurea in disease modification [13,21]. A study by Guillaume showed that the amount of fetal hemoglobin has a direct effect in reducing the morbidity and mortality associated with SCD and episodes of painful crisis. However, its role is limited once a painful event has occurred [22,23]. In our study, two-thirds of patients presented with pain affecting multiple sites, in contrast to observations in the study conducted in Uganda, where most patients had pain in their extremities [6]. Another study by Linda Frank indicated that children with diffuse pain had longer periods of hospital stay with no association with pain scales [9,24]. The most prevalent region of pain in Linda’s study was the lower back, followed by the abdomen, legs, and chest, while in this study, the majority of patients had pain in multiple sites [9]. A study by McClish found that pain located on the chest was more commonly associated with hospital admissions than pain in other sites. This was due to the high likelihood of having acute chest syndrome [25].

The majority of those who had inadequate pain were aged above 14 years. This was comparable to a study conducted in the UK, which observed that the VOC rate and other complications tend to gradually rise with advancing age in SCD patients. Another study found that older children had longer painful episodes, with no changes in frequency or intensity [26,27,28]. We observed that the incidence of inadequate pain control in older age was higher than that in younger age in univariate analysis. This result might be due to the difference in the tools used to allocate pain scores between young and older individuals. Our study did not find an association between age and an increase in the frequency of pain crises. Despite the absence of direct association in this study, other studies have shown that pain episodes increase with age and are more perceived in adolescents than in young children. In the majority of pediatric patients, pain is reported by caretakers rather than patients themselves [10].

Our study had several limitations. First, recall bias may have affected the reported pain episodes, as most of the pain information was patient-reported. Secondly, we were unable to establish the causes of inadequate pain control, as the study focused on ascertaining the factors affecting pain control. Also, the study was limited by the inability to assess changes in variables over time. Future studies should evaluate the adequacy of pain control in primary and tertiary-level healthcare facilities in a prospective cohort of SCD patients.

## 5. Conclusions

One-third of patients with SCD at tertiary healthcare facilities in Dar es Salaam reported suboptimal pain control. The significantly associated factors were residing outside Dar es Salaam, receiving initial pain management from other healthcare facilities, presenting to the hospital with moderate to severe pain, and having a fever. Further research is warranted to elucidate ways of optimizing the management of pain in patients with SCD in Tanzania.

## Figures and Tables

**Table 1:** Baseline social-demographic and clinical characteristics of the study participants.

Variable	Mean ± SD	Frequency (%), *N* = 390
**Age (years)**	15 ± 6	
≤14		230 (59.0)
>14		160 (41.0)
**Residence**		
Dar es Salaam		224 (57.4)
Outside Dar es Salaam		166 (42.6)
**Hb—Steady state (g/dL)**	7.6 ± 1	
**Hb on recruitment (g/dL)**		
Below steady state		35 (9.0)
Steady state		355 (91.0)
**Number of pain episodes per year**		
<3		304 (77.9)
≥3		86 (22.1)
**Pain scale**		
Mild pain (Level 1–3)		252 (64.6)
Moderate pain (Level 4–7)		103 (26.4)
severe pain (Level 8–10)		35 (9.0)
**Hydroxyurea use**		
Yes		243 (62.3)
No		147 (37.7)
**Body temperature (°C)**		
≤37.6 °C		374 (95.8)
>37.5 °C		16 (4.2)

**Table 2:** Factors associated with inadequate pain control among patients with SCD who presented with pain.

Variable	Adequacy of Pain Control		*p*-Value
	Adequate	Inadequate	
**Age (Years)**			
≤14	168 (73.0)	62 (27.0)	
>14	101 (63.1)	59 (36.9)	**0.04**
**Gender**			
Male	134 (69.8)	58 (30.2)	
Female	135 (68.6)	63 (31.8)	0.74
**Education**			
Primary or below	160 (71.1)	65 (28.9)	
Secondary or above	109 (66.1)	56 (33.9)	0.32
**Residence**			
Dar es Salaam	170 (75.9)	54 (24.1)	
Outside Dar es Salaam	99 (59.6)	67 (40.4)	**0.001**
**Recruitment Hb (g/dL)**			
Below steady state	22 (62.9)	13 (37.1)	
Steady state	247 (69.6)	108 (30.4)	0.45
**Pain episodes per year**			
<3	209 (68.8)	95 (31.3)	
≥3	60 (69.8)	26 (30.2)	0.89
**Pain scale**			
Mild pain (Levels 1–3)	190 (75.4)	62 (24.6)	
Moderate pain (Levels 4–5)	49 (47.6)	54 (52.4)	
Severe pain (Levels 6–10)	30 (85.7)	5 (14.3)	**<0.001**
**Site of maximum pain**			
Extremities	17 (77.3)	5 (22.7)	
Head and neck	89 (63.1)	52 (36.9)	
Multiple sites	110 (72.8)	41 (27.2)	
Trunk	53 (69.7)	23 (30.3)	0.3
**Hydroxyurea use**			
Yes	176 (72.4)	67 (27.6)	
No	93 (63.3)	54 (36.7)	0.07
**Fever (≥37.5 °C)**			
No	261 (69.8)	113 (30.2)	
Yes	8 (50.0)	8 (50.0)	0.09
**Hydration status**			
Well hydrated	254 (69.8)	110 (30.2)	
Dehydrated	15 (57.7)	11 (42.3)	0.19
**Site of initial pain management**			
Home	75 (68.2)	35 (31.8)	
Other healthcare facilities	76 (51.0)	73 (49.0)	
MNH/MRRH	118 (90.1)	13 (9.9)	**<0.001**
**Steady HB (g/dL)**			
≤7	163 (68.8)	74 (31.2)	
>7	106 (69.3)	47 (30.7)	1.00

**Table 3:** Multivariable logistic regression for the factors associated with inadequate pain control.

Variables	Crude OR	*p*-Value	Adjusted OR (95% CI)	*p*-Value
**Age (years)**				
≤14	Ref		Ref	
>14	1.6 (1–2.4)	0.04	1.3 (0.8–2.1)	0.36
**Residence**				
Dar es Salaam	Ref		Ref	
Outside Dar es Salaam	2.1 (1.4–3.3)	0.01	1.74 (1.1–2.9)	**0.03**
**Pain scale**				
Mild pain	Ref		Ref	
Moderate pain	3.4 (2–5.5)	<0.001	2.2 (1.3–3.8)	**0.006**
Severe pain	0.5 (0.2–1.4)	0.18	0.33 (0.1–1.0)	**0.04**
**Hydroxyurea use**				
Yes	Ref		Ref	
No	1.5 (1.0–2.4)	0.07	1.42 (0.9–2.4)	0.18
**Fever**				
No	Ref		Ref	
Yes	2.3 (0.8–6.5)	0.1	3.8 (1.1–13.9)	**0.04**
**Site of initial pain management**				
Home	Ref	Ref	Ref	
Other healthcare facility	2 (1.2–3.4)	0.006	2.5 (1.5–4.5)	**0.001**
MRRH/MNH	0.3 (0.1–0.5)	<0.01	0.29 (0.1–0.6)	**<0.001**

## Data Availability

The data processed in this study are available on request from the corresponding author due to privacy reasons.
